# NDRG2 promotes adriamycin sensitivity through a Bad/p53 complex at the mitochondria in breast cancer

**DOI:** 10.18632/oncotarget.16035

**Published:** 2017-03-09

**Authors:** Yifang Wei, Shentong Yu, Yongping Zhang, Yuan Zhang, Huadong Zhao, Zhixiong Xiao, Libo Yao, Suning Chen, Jian Zhang

**Affiliations:** ^1^ State Key Laboratory of Cancer Biology, Department of Biochemistry and Molecular Biology, The Fourth Military Medical University, Xi’an, 710032, Shaanxi, China; ^2^ Department of Pathology, The Fourth Military Medical University, Xi’an, 710032, Shaanxi, China; ^3^ Life Sciences Institute and Innovation Center for Cell Signaling Network, Zhejiang University, 310058, Hangzhou, China; ^4^ Department of Oncology, The State Key Discipline of Cell Biology, Xijing Hospital, The Fourth Military Medical University, Xi’an, 710032, Shaanxi, China; ^5^ Department of General Surgery, Tangdu Hospital, The Fourth Military Medical University, 710038, Xi’an, China; ^6^ College of Life Science, Sichuan University, Chendu, 610065, Sichuan, China; ^7^ Department of Pharmacy, Xijing Hospital, The Fourth Military Medical University, Xi’an, 710032, Shaanxi, China

**Keywords:** NDRG2, P53, Bad, chemotherapy resistance, breast cancer

## Abstract

Chemo-resistance presents a difficult challenge for the treatment of breast cancer. Our previous study showed that N-Myc downstream-regulated gene 2 (NDRG2) is involved in p53-mediated apoptosis induced by chemotherapy, through a mechanism that has so far remained obscure. Here, we explored the role of NDRG2 in chemo-resistance with a focus on Adriamycin (ADR) and found that NDRG2 expression decreased in ADR resistance breast cancer cells. Interestingly, NDRG2 can promote ADR sensitivity by inhibiting proliferation, enhancing cellular damage responses, and promoting apoptosis in a p53-dependent manner. We also found that NDRG2 could upregulate Bad expression by increasing its half-life, which is associated with p53 to mitochondria. Hence, our collective data provided the first evidence that NDRG2 promoting sensitivity of breast cancer is dependent on p53 by preventing p53 from entering the nucleus rather than changing its expression.

## INTRODUCTION

Breast cancer is the most frequently diagnosed tumors and the leading cause of cancer-related mortality among females worldwide [1]. Chemotherapeutics has been applied in clinical breast cancer treatment maturely, whereas, the intractable problem of drug sensitivity obstruct its expected efficiency. In recent decades, various mechanisms of drug resistance have been proposed, including miRNAs such as miR-218[2] and miR-205[3], ATP-Binding Cassette (ABC) transporters [4], such as P-glycoprotein (ABCB1) [5, 6] and ABCG2[7], and tumor suppressor genes such as Rb [8] and p53[9, 10]. Among them, the function of p53 in drug sensitivity of breast cancer is widely studied.

The tumor suppressor p53 is a key player in the cellular response to DNA damage induced by chemotherapy, making it one of the most comprehensively studied molecules in cancer research. The p53 can be activated by DNA damage to promote cell-cycle checkpoints, DNA repair, and apoptosis. As a transcription factor, p53 can induce apoptosis by trans-activating numerous target genes in the apoptotic pathway. Moreover, p53 can promote apoptosis through transcription-independent mechanisms by translocation to mitochondria [11, 12]. However, the potential effect of p53 on the development of chemotherapy resistance has not been well explored.

N-Myc downstream-regulated gene 2 (NDRG2), which belongs to the NDRG family, was firstly cloned by our laboratory from a normal human brain cDNA library (Gene Bank, Accession No. AF 159092) [13]. NDRG2 has been found decreased or null expressed in breast cancer and other tumor tissues [14, 15]. NDRG2 also inhibited proliferation and promoted cell apoptosis in many malignant tumors [16, 17]. In our previous study, we found that NDRG2 can be transcriptionally activated by p53 and involved in p53-mediated apoptosis pathway [18]. However, the molecular mechanism of NDRG2 in p53-mediated apoptosis, especially in breast cancer, remains unclear.

In our study, we demonstrated that NDRG2 could promote drug sensitivity of breast cancer cells in a p53-dependent manner. By analyzing the response of ADR-treated breast cancer cells, we found that overexpression of NDRG2 could inhibit proliferation, enhance DNA damage response, and promote apoptosis, all of which suggests the great potential of NDRG2 in increasing drug sensitivity of breast cancer cells. In addition, we explored the mechanism of NDRG2 in promoting drug sensitivity and found that NDRG2 could inhibit Bad degradation by increasing its protein stability. Moreover, excessive Bad can interact with p53 at the mitochondria, which induces apoptosis through transcription-independent mechanism of p53. Simultaneously, cytosolic p53 is detained by the excessive Bad, thus preventing p53 from entering the nucleus for further transcription of DNA damage repair genes.

## RESULTS

### NDRG2 expression is suppressed in ADR-resistant breast cancers

**Figure 1:**
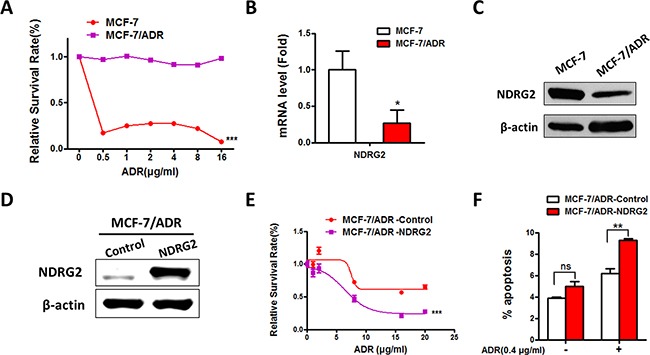
Expression of NDRG2 in MCF-7/ADR and MCF-7 cells and the effect of NDRG2 in ADR sensitivity **(A)** Cell viability assays in MCF-7 and MCF-7/ADR cells in the presence of a range of ADR concentrations. **(B)** The mRNA level of NDRG2 was confirmed by quantitative RT-PCR. **(C)** WB representing NDRG2 in MCF-7 and MCF-7/ADR cells. GAPDH was used as a loading control. **(D)** WB representing NDRG2 in MCF-7/ADR-Control and MCF-7/ADR-NDRG2 cells. GAPDH was measured as a loading control. **(E)** Cell viability assays in MCF-7/ADR-Control and MCF-7/ADR-NDRG2 cells in the presence of a range of ADR concentrations. **(F)** AnnexinV/PI apoptosis assays were performed in MCF-7/ADR-Control and MCF-7/ADR-NDRG2 cells in the presence of a range of ADR concentrations.

To investigate whether NDRG2 was involved in the process of acquiring ADR resistance of breast cancer cells, qRT-PCR and Western blot (WB) analyses were performed to examine the effects of endogenous NDRG2 expression in MCF-7/ADR and MCF-7 cells (Figure 1A). Both NDRG2 mRNA and protein levels were decreased in MCF-7/ADR cells compared with MCF-7 cells (Figure 1B and 1C), indicating that NDRG2 might regulate the ADR resistance of breast cancer. To better evaluate the function of NDRG2 in ADR response, we established the stable cell lines of NDRG2 overexpression in MCF-7/ADR cells based on lentivirus system (MCF-7/ADR-NDRG2). It is demonstrated that the expression of NDRG2 was dramatically increased at the protein levels in MCF-7/ADR cells (Figure 1D). And when cells were treated with increasing amounts of ADR, overexpression of NDRG2 in MCF-7/ADR cells could obviously increase the cytotoxic effect of ADR compared with MCF-7/ADR cells (Figure 1E). We further used Annexin V/PI staining to identify the apoptotic cells and found that NDRG2 overexpression significantly increased apoptotic cells compared with the control group (Figure 1F). Taken together, our data showed that NDRG2 could increase ADR sensitivity in breast cancer MCF-7 cells.

### NDRG2 promotes ADR sensitivity in breast cancer in a p53-dependent manner

**Figure 2:**
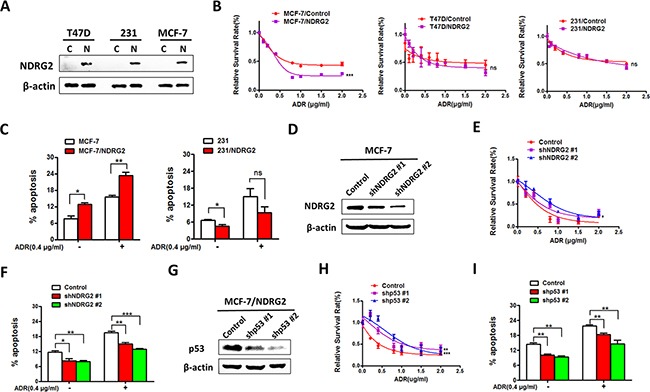
NDRG2 overexpression promotes ADR sensitivity in p53wt but not in p53 mutant cells **(A)** WB representing NDRG2 in indicated cells. β-actin was used as a loading control. **(B)** Cell viability assays or AnnexinV/PI apoptosis assays. **(C)** were performed in indicated cells in the presence of a range of ADR concentrations. **(D)** WB representing NDRG2 in indicated cells. β-actin was used as a loading control. **(E)** Cell viability assays or AnnexinV/PI apoptosis assays **(F)** were performed in indicated cells in the presence of a range of ADR concentrations. **(G)** WB represening p53 in indicated cells. β-actin was used as a loading control. **(H)** Cell viability assays or AnnexinV/PI apoptosis assays **(I)** were performed in indicated cells in the presence of a range of ADR concentrations.

To further investigate the effect of NDRG2 in ADR resistance of breast cancer cells, we overexpressed NDRG2 in breast cancer cells with different p53 status, two mutant p53 cell lines: T47D (L194F) and MDA-MB-231(R280K), and one wildtype p53 cell line MCF-7 (Figure 2A). MTT assay was performed to measure cell viability with ADR treatment. The results showed that NDRG2 could significantly inhibit the proliferation of MCF-7 cells under ADR treatment (Figure 2B). However, NDRG2 could not alter the drug sensitivity in two mutant p53 cells (Figure 2B). As the DNA damage response functions, followed by ADR treatment, if the broken DNA cannot be repaired efficiently and accurately, it will finally result in cell apoptosis or death. To assess the contribution of NDRG2 to the apoptosis in drug sensitivity of breast cancer cells, we applied different methods with various mechanisms to evaluate this response. Annexin V/PI assay showed that NDRG2 overexpression in MCF-7 cells significantly increased apoptotic cells compared with the control group with the presence or absence of ADR. Whereas in the p53 mutant MDA-MB-231 cell line, when treated with ADR to induce DNA damage, no obvious apoptotic difference was observed in the NDRG2 overexpression group compared with the control (Figure 2C). Both transmission electron microscopy (TEM) and TUNEL analysis showed that NDRG2 overexpression in MCF-7 cells could increase apoptotic cells, which is consistent with the results of Annexin V/PI assay. However, in MDA-MB-231 cells, NDRG2 decreased the apoptotic cells (Supplementary Figure 1, 2 and 3). We further knockdown NDRG2 by lentivirus infection in MCF-7 cells (Figure 2D). MTT assay and Annexin V/PI assay demonstrated the consistence with the promoting effect of NDRG2 overexpression on ADR sensitivity, NDRG2 knockdown in MCF-7 cells inhibits ADR sensitivity (Figure 2E and 2F).

To further verify whether the effect of NDRG2 on promoting drug sensitivity depends on p53, we used lentivirus-mediated shRNA to further knockdown p53 in MCF-7/NDRG2 cells (Figure 2G) and then measured cell viability and apoptosis. The results showed that in MCF-7/NDRG2 cells, p53 knockdown did promote proliferation and inhibit the apoptosis induced by NDRG2 (Figure 2H and 2I). This results suggested that knockdown p53 in MCF-7 cells compromised the pro-apoptotic function of NDRG2. Thus, our data provided strong evidences that NDRG2 promotes ADR sensitivity dependent on p53.

### NDRG2 aggravates DNA damage response in p53wt but not in p53mut cells

**Figure 3:**
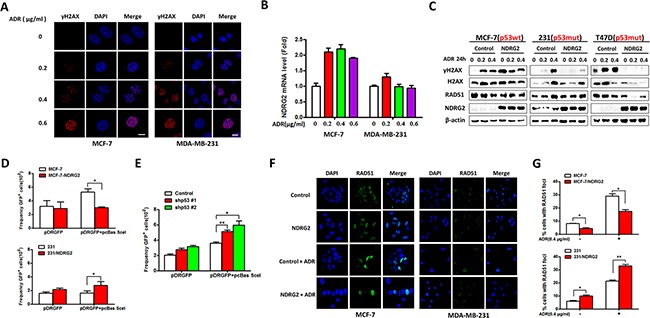
NDRG2 overexpression enhances DNA damage **(A)** Indicated cells were stained for γ-H2AX. DAPI was used for nuclear staining. **(B)** The mRNA levels of NDRG2 in MCF-7 and MDA-MB-231 cells were confirmed by quantitative RT-PCR. **(C)** WB representing the indicated proteins in indicated cells. β-actin was used as a loading control. **(D)** and **(E)** Indicated cells were used to analyze I-SceI-induced HR. **(F)** Indicated cells were stained for RAD51. DAPI was used for nuclear staining. **(G)** The percentage of cells with different patterns divided by the number of foci.

To further examine the relationship between NDRG2 expression and DNA damage response, the expression level of phosphorylated histone H2AX (γ-H2AX), one of the DNA repair markers, was tested by immunostaining and western blotting. As showed in Figure 3A and 3B, in MCF-7 cells, the number of γ-H2AX foci formation and NDRG2 mRNA levels increased with the increasing dose of ADR. However, in MDA-MB-231 cells, differences in NDRG2 mRNAs among different groups were not detected. We further examined the protein expression of γ-H2AX and RAD51 by western blotting. In the MCF-7/Control cells, the γ-H2AX significantly increased with increasing dose of ADR, while the expression of γ-H2AX increased and then decreased in the presence of ADR in MCF-7/NDRG2 cells. In p53 mutant cells, the level of γ-H2AX increased with the ascending dose of ADR. This increase in γ-H2AX expression became less apparent and even disappeared in NDRG2 overexpression cells (Figure 3C).

To further examine the role of NDRG2 on the homologous recombination (HR) repair mechanism, we expressed the recombination substrate pDR-GFP for HR analysis in cells, and confirmed the GFP positive cells with I-SceI-induced DSB repair by flow cytometry [19]. The results showed that compared with the control group, NDRG2 overexpression caused dramatic alteration of I-SceI-induced HR frequency, with two folds decrease in MCF-7 cells and two folds increase in MDA-MB-231 cells, respectively (Figure 3D). We also performed the HR analysis in MCF-7/NDRG2 cells with p53-knockdown, and found that NDRG2 overexpression did not inhibit the HR efficiency after p53 abrogation (Figure 3E). Our data suggested that NDRG2 could attenuate the homologous recombination efficiency under ADR treatment, but the display of its functions on HR is surely dependent on p53. RAD51 foci formation was also tested by immunostaining. MCF-7/NDRG2 cells showed a decrease in the number of cells with RAD51 foci formation compared with control cells, which indicated that in response to DNA damage, NDRG2 could reduce the efficiency of RAD51 foci formation in MCF-7 cells. In contrast, NDRG2 increased RAD51 foci formation in p53 mutant cell line MDA-MB-231 with the presence or absence of ADR (Figure 3F and 3G).

Collectively, these data showed that NDRG2 expression could promote DNA damage response induced by ADR, and thus sensitized breast cancer cells to chemotherapeutic agents in a p53-dependent manner.

### NDRG2 exerts its pro-apoptotic effects by improving Bad expression

**Figure 4:**
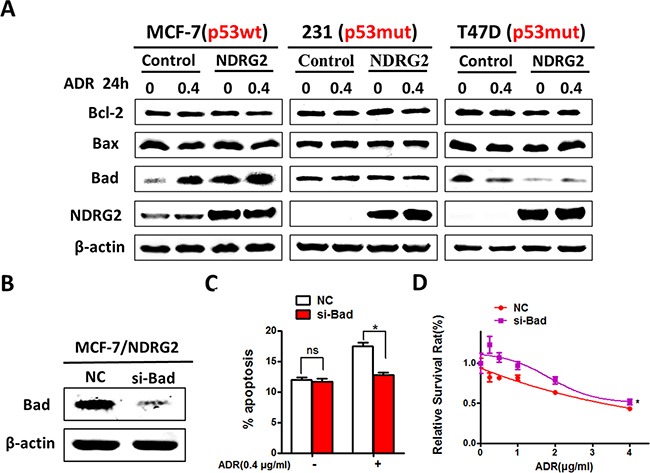
NDRG2 exerts its apoptotic effects by improving Bad expression **(A)** WB representing the indicated proteins in cells. β-actin was used as a loading control. **(B)** WB representing Bad in MCF-7/NDRG2 cells transfected with Bad siRNA or control siRNA. β-actin was used as a loading control. **(C)** AnnexinV/PI apoptosis assays were performed in MCF-7 cells transfected with Bad siRNA or control siRNA in the presence and absence of ADR. **(D)** Cell viability assays in MCF-7 cells transfected with Bad siRNA or control siRNA in the presence of a range of ADR concentrations.

So far, our data provided strong evidence about function of NDRG2 on promoting drug sensitivity. To further explore the mechanism, we tested apoptosis related Bcl-2 family proteins by western blotting, and found that NDRG2 overexpression had no effect on expression of Bax and Bcl-2 in those three cell lines. Interesting, NDRG2 induced the expression of the pro-apoptotic protein Bad in MCF-7 cells with or without ADR. Consistent with the results of apoptosis, NDRG2 could not change the expression levels of Bad in T47D cell line. Simultaneously, NDRG2 decreased Bad expression in MDA-MB-231 cells (Figure 4A). These results indicated that DNA damage could induce increased expression of Bad by NDRG2, which was p53 dependent. We further investigated the role of Bad in NDRG2-induced apoptosis by using siRNA to suppress the endogenous Bad (Figure 4B). The data showed that knockdown of Bad decreased the percentage of apoptosis induced by NDRG2, and blocked the ADR sensitivity in NDRG2-overexpressed cells (Figure 4C and 4D).

### NDRG2 regulates the protein stability of Bad

**Figure 5:**
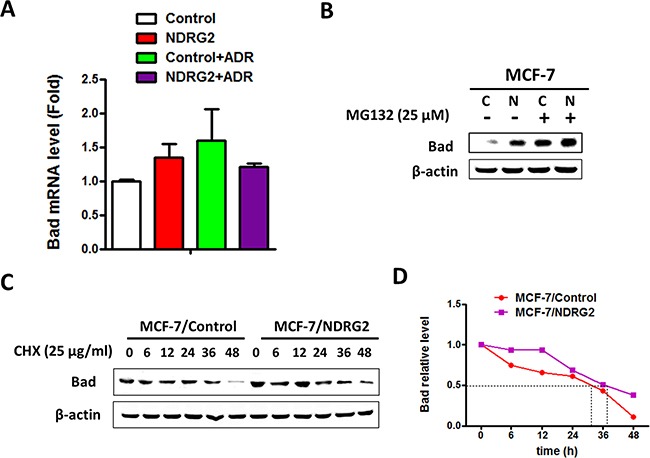
NDRG2 regulates the protein stability of Bad **(A)** Bad mRNA levels in MCF-7/Control and MCF-7/NDRG2 cells in the presence and absence of ADR. β-actin served as control. **(B)** WB representing Bad in MCF-7/Control and MCF-7/NDRG2 cells treated with 25 μM MG132 for 4 hrs. β-actin served as control. **(C)** WB representing the indicated proteins in MCF-7/Control and MCF-7/NDRG2 cells treated with CHX (25 μg/ml). β-actin served as control. **(D)** WB bands of Bad were quantified by the ImageJ software for calculating the half-life of Bad.

We further elucidated the underlying mechanism of how NDRG2 regulating Bad expression. Bad mRNA level was tested by RT-qPCR and results showed that NDRG2 did not change the mRNA level of Bad, indicating that it might be through protein stability regulation (Figure 5A). MG132, a potent inhibitor of the proteasome, significantly increased Bad expression levels and enhanced upregulation effect of NDRG2, suggesting that NDRG2 counteracts Bad degradation (Figure 5B). Furthermore, expression levels of Bad protein were analyzed by western blotting and quantified by the ImageJ software for calculating the half-life. Results revealed that NDRG2 overexpression prolonged the half-life of Bad (Figure 5C-5D). Taken together, we have identified that NDRG2 could increase Bad stability to promote apoptosis of breast cancer cells.

### NDRG2 overexpression attenuates p53 nuclear translocation

**Figure 6:**
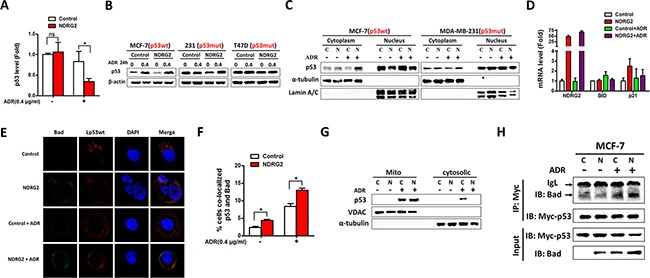
NDRG2 overexpression attenuates p53 nuclear translocation **(A)** The p53 mRNA levels in MCF-7/Control and MCF-7/NDRG2 cells in the presence and absence of ADR. β-actin served as control. **(B)** WB representing p53 in indicated cells in the presence and absence of ADR. β-actin was used as loading control. **(C)** WB representing nuclear and cytoplasmic p53 in indicated cells in the presence and absence of ADR. α-tubulin and Lamin A/C were used as cytoplasm and nucleus loading control respectively. **(D)** The mRNA levels of NDRG2, Bid and p21 in MCF-7/Control and MCF-7/NDRG2 cells in the presence and absence of ADR. β-actin served as control. **(E)** MCF-7/Control and MCF-7/NDRG2 cells transfected with FLAG-Lp53wt were stained by FLAG and Bad antibodies in the presence and absence of ADR. DAPI was used for nuclear staining. **(F)** Percentages of cells co-localized p53 and Bad were analyzed from three independent experiments. **(G)** WB representing mitochondrial and crude p53 in MCF-7/Control and MCF-7/NDRG2 cells in the presence and absence of ADR. α-tubulin and VDAC were used as crude and mitochondria loading control respectively. **(H)** p53 and Bad were coimmunoprecipitated from MCF-7 cells. Cells were lysed, immunoprecipitated with c-Myc tag antibody and immunoblotted with Bad antibodies.

To further verify the mechanism of NDRG2 in promoting drug sensitivity in a p53-dependent manner, we evaluated how NDRG2 regulating p53 function. RT-qPCR suggested that p53 mRNA level was not affected by NDRG2 in the absence of ADR. Following drug treatment, p53 mRNA was reduced and this reduction became even less apparent with NDRG2 overexpression (Figure 6A). Western blot results showed that NDRG2 overexpression has no effect on p53 protein level in the presence and absence of ADR (Figure 6B). Subsequently, we analyzed whether NDRG2 affected the subcellular localization of p53, nuclear and cytosolic extracts were prepared for western blot analysis. When cells were treated with ADR, cytosolic p53 was dramatically increased in MCF-7/NDRG2 cells compared with the control group. However, NDRG2 had no effect on the distribution of p53 in MDA-MB-231 cells (Figure 6C). Our data showed that although p53 expression levels was increased in MCF-7/NDRG2 cells under ADR treatment, downstream targets of p53 might not be activated through transcriptional regulation. Herein, we used RT-qPCR analysis and found that numerous p53-targeting genes including Bad, Bid, p21 mRNAs remain unchanged with NDRG2 overexpression (Figure 5A and 6D), which is consistent with the cytosolic localization of p53.

Previous data suggested that Bad may bind to p53 in the cytoplasm, specifically at the mitochondria and then promoted apoptosis [11]. We hypothesized that NDRG2 overexpression could promote Bad/p53 complex at the mitochondria. Hence cells were transfected with Lp53wt which harbored tag protein FLAG and specifically expressed p53 at the mitochondria [12]. Then the fluorescence distribution patterns were compared. The results showed that Lwtp53 was undetectable within nuclei. After NDRG2 overexpression, the level of endogenous Bad increased, and also was detected in many large cytoplasmic speckles that were stained by the FLAG antibody as well (Figure 6E and 6F). At the same time, cytosolic and mitochondrial extracts were prepared for western blot analysis. Results showed that NDRG2 overexpression and ADR treatment made an increasing accumulation of p53 in the mitochondrial fraction, with a corresponding decrease in their cytosolic levels (Figure 6G). Furthermore, we performed co-immunoprecipitation experiments to test whether p53 physically interacted with Bad. The result showed that NDRG2 could enhance the interaction between Bad and p53 under ADR treatment (Figure 6H). Our data for the first time demonstrated that NDRG2 could promote the accumulation of p53 on the mitochondria by Bad/p53 complex and trigger apoptosis to sensitize breast cancer to ADR.

## DISCUSSION

Chemotherapy is widely used to treat almost all types of cancer. Yet the response to chemotherapy is often varying. To a specific chemotherapy regimen, generally only a fraction of patients achieve full response, while others respond poorly and only suffer from side effects, making it a major obstacle to effective cancer treatment. In this study, we uncovered the novel function of NDRG2 in response to chemotherapy drugs with a focus on ADR. Our experiments demonstrated for the first time that tumor suppressor NDRG2 was significantly decreased in MCF-7/ADR cells, and NDRG2 overexpression could improve breast cancer cells sensitivity to ADR. Furthermore, under ADR treatment, NDRG2 could promote apoptosis and DNA damage response as well as inhibit cell proliferation. But all those functions of NDRG2 depended on wild type p53 states. More importantly, NDRG2 induced Bad expression by promoting its stability. Then excessive Bad interacted with p53 at the mitochondria to induce apoptosis, which inhibited the nuclear translocation of p53.

The *TP53* gene is the most frequently mutated gene in human cancers, such as in ovarian (50%), colorectal (43%) and breast cancers (23%) [20, 21]. Mutant p53 lost the role of tumor suppressor and showed a gain of function (GOF), such as a role in cell reprogramming and expansion. What's more, mutant p53 was correlated with chemotherapy resistance and poor prognosis in breast cancer as well as several other cancer types [22]. In our study, we used two mutant breast cancer lines MDA-MB-231 (R280K) and T47D (C194T), which harboring p53 mutant in DNA banding domain. Although, mutant p53 can enhance invasion and motility in those two cells [23, 24], there was no evidence showing the role of mutant p53 in chemotherapy response in those two cells.

It has been proposed that p53 has dual mechanisms for inducing cell death. As a transcription factor, p53 induces transcription of numerous cell cycle regulators and pro-apoptotic genes as well as repressing the transcription of anti-apoptotic proteins [25, 26]. Alternatively, p53 can also promote cell death in a transcription-independent manner [27, 28]. It was observed that in response to stress, a fraction of p53 rapidly translocating to the cytoplasm and mitochondria, triggering mitochondrial outer membrane permeabilization [29, 30]. Studies have showed that p53 can translocate to the mitochondria by direct fusion with the mitochondrial import leader peptide of human ornithine transcarbamylase, such as Tid1, RECQL4, or the transmembrane domains of anti-apoptotic proteins Bcl-xl or Bad [11, 12, 31, 32]. In our study, when treated with NDRG2, Bad expression was upregulated, which in turn promoting p53 localization to the mitochondria and apoptosis induction.

**Figure 7:**
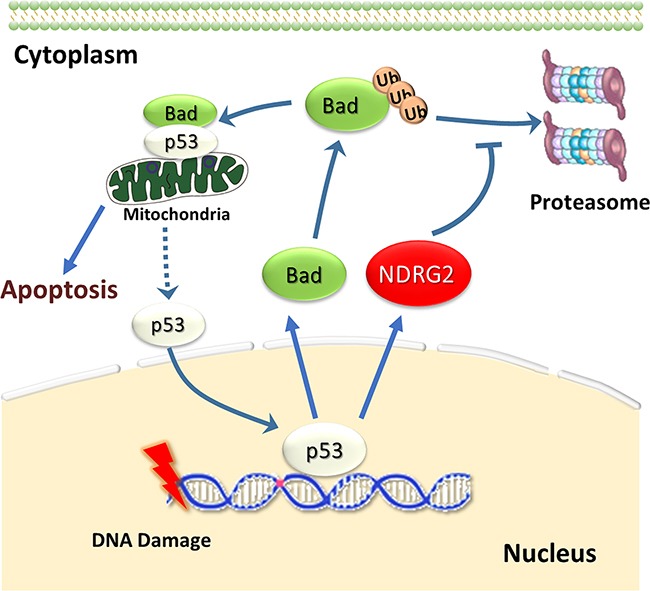
NDRG2 prevents p53 from entering nucleus by Bad/p53 complex Upon treatment with a DNA damaging agent such as ADR, NDRG2 and Bad gene expression can be trans-activated by p53. Simultaneously, NDRG2 could increase the protein stability of Bad, and detain p53 in mitochondria. Thus NDRG2 might prevent p53 from entering nucleus through enhancing Bad/p53 complex, which finally increasing the cellular sensitivity to ADR.

In summary, our results demonstrated that NDRG2 could promote chemotherapy sensitivity in breast cancer. As showed in Figure 7, we proposed a scheme upon DNA damage response, NDRG2 can upregulate Bad expression by increasing its half-life, which further promotes the formation of Bad/p53 complex at the mitochondria. Hence, our collective data for the first time suggest that NDRG2 promoting ADR sensitivity in breast cancer is a p53-dependent manner through preventing p53 from entering into the nucleus rather than changing its expression.

## MATERIALS AND METHODS

### Cell culture

Human breast cancer cell lines MCF-7, MDA-MB-231 and T47D cells were obtained from ATCC. MCF-7 cells with ADR resistance (MCF-7/ADR) were gift from Dr. Yu zuoren (TongJi university, Shanghai). MCF-7 cells were cultured in DMEM supplemented with 0.01 mg/ml bovine insulin, MDA-MB-231cells were cultured in DMEM, T47D cells were maintained in RPMI 1640, supplemented with 10% fetal bovine serum. All three cell lines were incubated in a humidified atmosphere of 5% CO_2_ at 37 °C.

### Cell fractionation and mitochondria isolation

Mitochondria from MCF-7 and MDA-MB-231 cells have been prepared using Cell Mitochondria Isolation Kit (Beyotime). Nuclear and cytoplasmic fractions were prepared using the Nuclear and Cytoplasmic Protein Extraction Kit (Beyotime) according to the manufacturer's instructions.

### qRT-PCR

For qRT-PCR, total RNA was prepared from cell lines. The results were normalized to the expression of β-actin, and the primer sequences were as follows: *NDRG*2 forward primer: *gagatatgctcttaaccacccg*, reverse primer: *gctgcccaatccatccaa*; P53 forward primer: *actgtac caccatccactacaact*, reverse primer: *acaaacacgcacctcaaagc*, p21 forward primer: *tacccttgtgcctcgctcag*, reverse primer: *gagaagatcagccggcgttt*, Bid forward primer: *ttaaagaatcctttgcggc*, reverse primer: *gtgattctcctgcttcag*, Bad forward primer: *ggaaacccggtggggccac*, reverse primer: *accagtagcgggtggtc*. qRT-PCR was performed on a C1000 thermal cycler (Bio-Rad, Hercules, CA)

### Co-immunoprecipitation

To immunoprecipitate the ectopically expressed Myc-tagged p53 proteins, transfected cells were lysed post-transfection in 500 μl lysis buffer (Beyotime). Subsequently, 1 mg protein lysates were incubated with anti-Myc antibodies and 30 μl Protein A-Agarose beads (all purchased from Santa Cruz Biotechnology) at 4°C overnight with gentle agitation. The beads were then pelleted and washed three times with lysis buffer. Immunoprecipitated proteins were eluted in 30 μl 2×SDS-PAGE sample loading buffer and incubated at 95°C for 5 min. The supernatants were subjected to SDS-PAGE and analyzed by immunoblotting.

### Western blot analysis

For Western blot analysis, total protein was prepared from cell lines. Immunoblotting were performed according to standard procedures with p53 (sigma and Cell signaling), Bad, Bax, Bcl-2, VDAC and NDRG2 (Cell Signaling), α-tubulin and β-actin (Boster) at room temperature for 1 hour and then at 4°C overnight. All blots were detected using the enhanced chemiluminescence (ECL) detection method.

### MTT assay

The 3-(4,5-dimethylthiazol-2-yl)-2,5-diphenyl-tetrazolium bromide (MTT) assays were performed as previously described. Briefly, cells were harvested from exponentially growing cultures, counted, and plated in 96-well plates at 1×10^4^ cells per well. Then cells were treated with a different concentration of ADR. After 72 hours, the cells were incubated with 5% MTT in PBS for 4 hours. The culture medium was then removed, and 150 μl DMSO was added. Optical density was measured at 490 nm.

### Homologous recombination assay

Homologous recombination analysis was done in cells expressing recombination substrate pDR-GFP according to previous publications [19]. Briefly, cells infected with lentivirus containing NDRG2 or control were co-transfected with pDR-GFP and I-Sce I expression vector, then GFP+ cells were sorted by flow cytometry.

### Gene transfection

To inhibit Bad expression, cells were seeded on 60 mm dishes, 24 hours later, cells were transfected using TurboFect Transfection Reagent (ThermoFisher Scientific, Wilmington, DE) and 60 nM of Bad siRNA (Gene-Pharma, Shanghai, China) or control siRNA according to the manufacturer's instructions

### Statistical analysis

For statistical analysis, Graph Pad Prism version 5c was used. All data shown are mean ± SEM of triplicate values from three separate experiments. Two treatment groups were compared using Student's t-test. P<0.05 was considered to be statistically significant.

This study was supported by National Natural Science Foundation of China (No. 81230043, 81421003 and 81372390).

## SUPPLEMENTARY MATERIALS FIGURES AND TABLES



## Abbreviations

NDRG2: N-Myc downstream regulated gene 2
ADR: Adriamycin
ABC: ATP-Binding Cassette
