# Supplementary material for: NDRG2 promotes adriamycin sensitivity through a Bad/p53 complex at the mitochondria in breast cancer
Source: Oncotarget. 2017 Mar 9;8(17):29038–47. doi: 10.18632/oncotarget.16035 (PMC5438710; doi:10.18632/oncotarget.16035)
Supplement: Supplementary file 1 [file oncotarget-08-29038-s001.pdf]

# NDRG2 promotes adriamycin sensitivity through a Bad/p53 complex at the mitochondria in breast cancer

## SUPPLEMENTARY MATERIALS

## SUPPLEMENTARY FIGURES

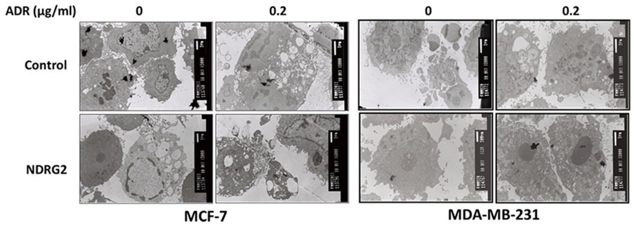


**Supplementary Figure 1: TEM micrographs of apoptosis were shown.**

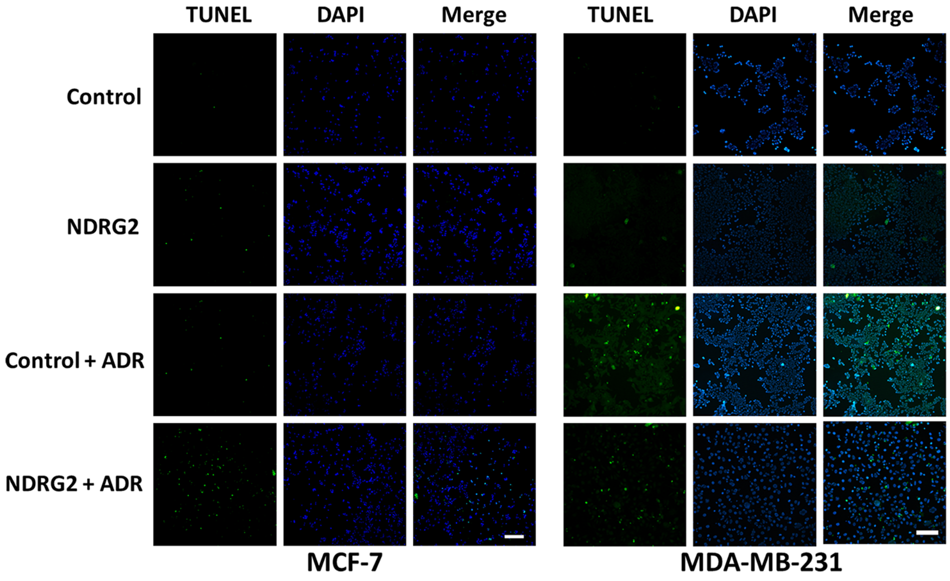


**Supplementary Figure 2: TUNEL assay were performed in indicated cells in the presence and absence of ADR.** DAPI was used for nuclear staining.

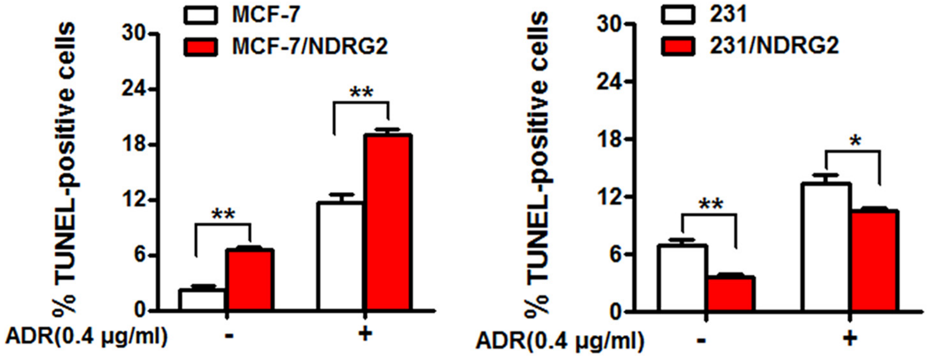


**Supplementary Figure 3: TUNEL assay were quantified for calculating the % TUNEL-positive cells.**
